# Reconstitution of the Costunolide Biosynthetic Pathway in Yeast and *Nicotiana benthamiana*

**DOI:** 10.1371/journal.pone.0023255

**Published:** 2011-08-15

**Authors:** Qing Liu, Mohammad Majdi, Katarina Cankar, Miriam Goedbloed, Tatsiana Charnikhova, Francel W. A. Verstappen, Ric C. H. de Vos, Jules Beekwilder, Sander van der Krol, Harro J. Bouwmeester

**Affiliations:** 1 Laboratory of Plant Physiology, Wageningen University, Wageningen, The Netherlands; 2 Agronomy and Plant Breeding Department, Faculty of Agriculture, University of Kurdistan, Sanandaj, Iran; 3 Plant Research International, Wageningen, The Netherlands; 4 Centre for BioSystems Genomics, Wageningen, The Netherlands; 5 Netherlands Metabolomics Centre, Leiden, The Netherlands; Instituto de Biología Molecular y Celular de Plantas, Spain

## Abstract

The sesquiterpene costunolide has a broad range of biological activities and is the parent compound for many other biologically active sesquiterpenes such as parthenolide. Two enzymes of the pathway leading to costunolide have been previously characterized: germacrene A synthase (GAS) and germacrene A oxidase (GAO), which together catalyse the biosynthesis of germacra-1(10),4,11(13)-trien-12-oic acid. However, the gene responsible for the last step toward costunolide has not been characterized until now. Here we show that chicory costunolide synthase (CiCOS), CYP71BL3, can catalyse the oxidation of germacra-1(10),4,11(13)-trien-12-oic acid to yield costunolide. Co-expression of feverfew *GAS* (*TpGAS*), chicory *GAO* (*CiGAO*), and chicory *COS* (*CiCOS*) in yeast resulted in the biosynthesis of costunolide. The catalytic activity of *TpGAS*, *CiGAO* and *CiCOS* was also verified *in planta* by transient expression in *Nicotiana benthamiana*. Mitochondrial targeting of *TpGAS* resulted in a significant increase in the production of germacrene A compared with the native cytosolic targeting. When the *N. benthamiana* leaves were co-infiltrated with *TpGAS* and *CiGAO*, germacrene A almost completely disappeared as a result of the presence of CiGAO. Transient expression of *TpGAS*, *CiGAO* and *CiCOS* in *N. benthamiana* leaves resulted in costunolide production of up to 60 ng.g^−1^ FW. In addition, two new compounds were formed that were identified as costunolide-glutathione and costunolide-cysteine conjugates.

## Introduction

Sesquiterpene lactones (SLs) are a major class of plant secondary metabolites. These bitter tasting, lipophilic molecules form the active constituents of a variety of medicinal plants used in traditional medicine [1], [2]. Some SLs show bioactivities which are beneficial to human health, such as anti-inflammatory (*e.g.* helenalin) [3], anti-cancer (*e.g.* costunolide) [4], and anti-malarial (artemisinin) [5]. The majority of SLs have been reported from the Asteraceae family, with over 4000 different SLs that have been identified [6]. While the detailed structure of those SLs varies, their backbones are constrained to a limited set of core skeletons, such as germacranolide, eudesmanolide and guaianolide [7], [8], [9]. For all these three types of sesquiterpene lactones costunolide is generally considered the common precursor [6]. Costunolide has been detected in many medicinal plants and several biological activities were ascribed to it including anti-carcinogenic, anti-viral, anti-fungal, and immunosuppressive activities [10], [11], [12], [13], [14]. Synthetic derivatives of costunolide such as 13-amino costunolide derivatives have anti-cancer activity [15] and also biosynthetic downstream products derived from costunolide have been reported to have interesting biological properties. For example, parthenolide has been reported to have anti-inflammatory and anti-cancer activity [16], [17].

**Figure 1:**
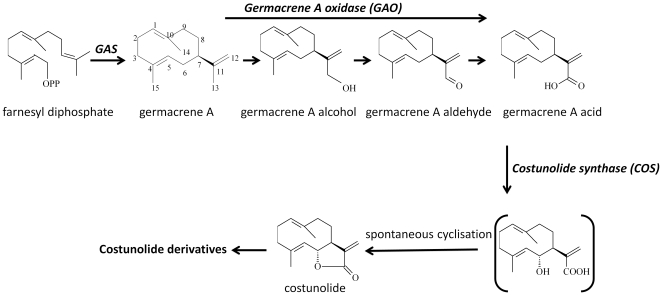
Biosynthetic pathway of costunolide in Asteraceae. *GAS*, germacrene A synthase.

Despite the importance of costunolide-derived SLs, the biosynthesis pathway of costunolide has not been fully elucidated. The pathway from FPP to costunolide was first proposed by de Kraker *et al.* based on the presence of enzymes in chicory roots that convert FPP to costunolide [6], [18], [19] (Figure 1). First, farnesyl diphosphate is converted to germacrene A by germacrene A synthase (GAS) [19]. GAS genes have been isolated and characterized from several members of the Asteraceae family, such as chicory [20], lettuce [21], *Artemisia annua* [22], and feverfew [23].

In the next step of the pathway, germacrene A is oxidized at its C13 methyl by germacrene A oxidase (GAO) to form germacra-1(10),4,11(13)-trien-12-ol, which is then further oxidised to germacra-1(10),4,11(13)-trien-12-al and germacra-1(10),4,11(13)-trien-12-oic acid [6], [24]. The C6 position of germacra-1(10),4,11(13)-trien-12-oic acid is subsequently hydroxylated by a putative cytochrome P450 mono-oxygenase, after which presumably spontaneous cyclization of the C6 hydroxyl and C12 carboxylic group leads to the formation of costunolide [6].

Although biosynthesis of costunolide from germacra-1(10),4,11(13)-trien-12-oic acid has been demonstrated in chicory biochemically [6], the corresponding gene responsible for this step has not been identified to date. It was shown that both germacrene A oxidase and costunolide synthase are cytochrome P450 enzymes. Recently, genes that encode germacrene A oxidase were cloned from a number of Asteraceae species [25]. A valencene oxidase gene (CYP71AV8) was also reported to have the germacrene A oxidase activity [26]. All these genes belong to the CYP71 group of cytochrome P450s. In the present study, we investigated 5 candidate CYP71 P450 genes from a chicory cDNA library for costunolide synthase activity. The putative *CiCOS* gene was characterised by reconstitution of the costunolide biosynthetic pathway in yeast as well as in *Nicotiana benthamiana*, and the products formed were analysed using GC-MS and LC-MS metabolic profiling.

## Results

### Optimizing germacrene A production *in planta*

**Figure 2:**
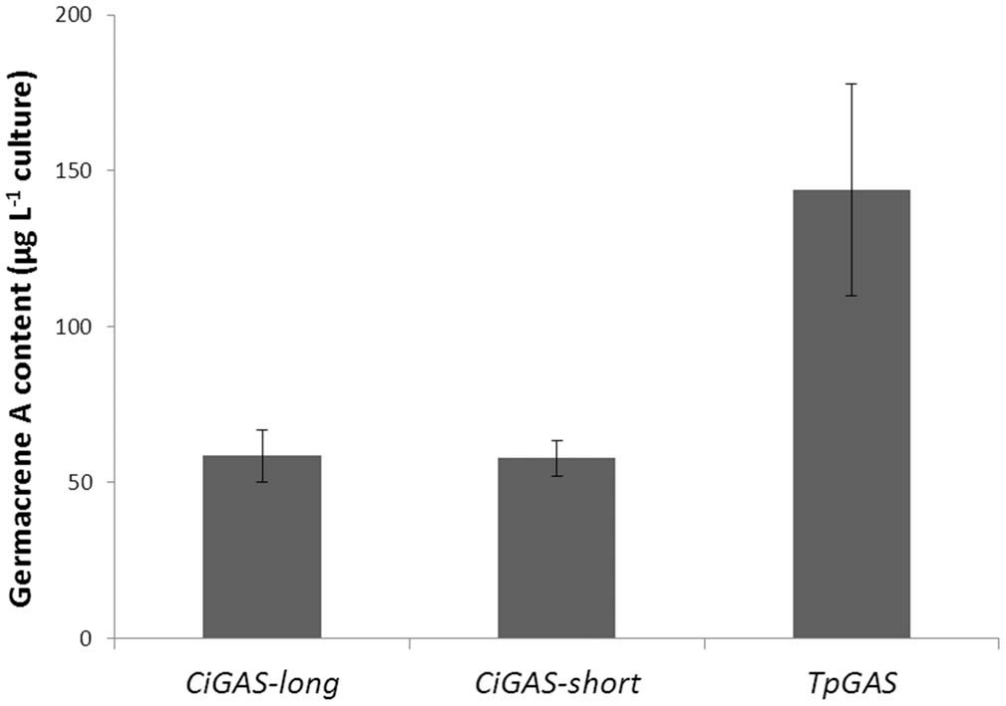
Germacrene A production in yeast. A yeast culture transformed by either *CiGAS-long*, *CiGAS-short* [20], or *TpGAS* [23]. Induced yeast culture medium was extracted and analysed by GC-MS.

**Figure 3:**
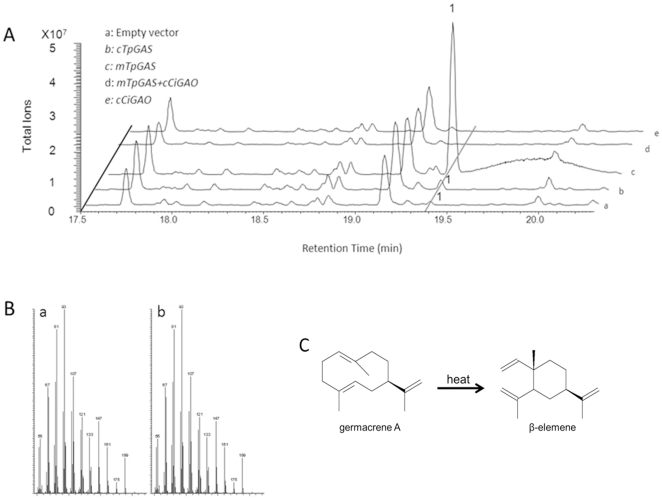
Headspace analysis of volatiles emitted from agro-infiltrated *Nicotiana benthamiana* leaves. A, GC-MS chromatograms are shown for the volatiles emitted from *N.benthamiana* leaves infiltrated with the indicated genes. Line a is a negative control, line b and c display the different amount of compound 1 (germacrene A) produced by *N. benthamiana* leaves infiltrated with *TpGAS* with different targeting signals: *mTpGAS*, mitochondrial targeting; c*TpGAS*, cytosolic targeting. Line d shows that compound 1 which is produced upon *mTpGAS* agro-infiltration disappears upon agro-infiltration with *CiGAO*. Agro-infiltration with *CiGAO* alone does not induce any volatile formation (Line e). B, the mass fragmentation patterns of compound 1 (a) and a β-elemene from the Wiley library (b). C, cope rearrangement of germacrene A to β-elemene by heat.

To produce germacrene A we used the *GAS* gene isolated from feverfew (*Tanacetum parthenium*), *TpGAS* [23]. After cloning of the full length coding sequence into a yeast expression vector, the TpGAS activity was compared with the previously characterized *GAS* genes from chicory (*CiGAS-l* and *CiGAS-s*) [20]. Results showed that yeast culture expressing *TpGAS* had an approximately three fold higher activity than that of the *CiGAS* gene(s) (Figure 2). Subsequently, the *TpGAS* cDNA - using its native targeting to the cytosol (*cTpGAS*) or equipped with a mitochondrial targeting signal (*mTpGAS*) - was cloned into a binary expression vector under control of the Rubisco promoter and introduced into *Agrobacterium tumefaciens*. For analysis of *in planta* activity, *N. benthamiana* leaves were agro-infiltrated with the *cTpGAS* or *mTpGAS* containing *A. tumefaciens* strain and were analysed after 3 days. In the headspace of *cTpGAS* agro-infiltrated *N. benthamiana*, germacrene A was detected while no germacrene A was detected in leaves infiltrated with the empty vector (Figure 3A, line a and b). It has been shown for several terpene synthases that targeting to the mitochondria rather than to the cytosol which is the native compartment for sesquiterpene synthases results in higher production, presumably because of higher substrate availability in the mitochondria [27], [28]. The *TpGAS* coding sequences was therefore fused to the CoxIV mitochondrial targeting sequence (*mTpGAS*). In *N. benthamiana* leaves infiltrated with *A. tumefaciens* carrying *mTpGAS*, the germacrene A production was approximately 15-fold higher than obtained by expression of *cTpGAS* (Figure 3A, line b and line c). Therefore, *mTpGAS* was used for the reconstruction of the costunolide pathway in *N. benthamiana*.

### Functional characterization of *GAO in planta*

**Figure 4:**
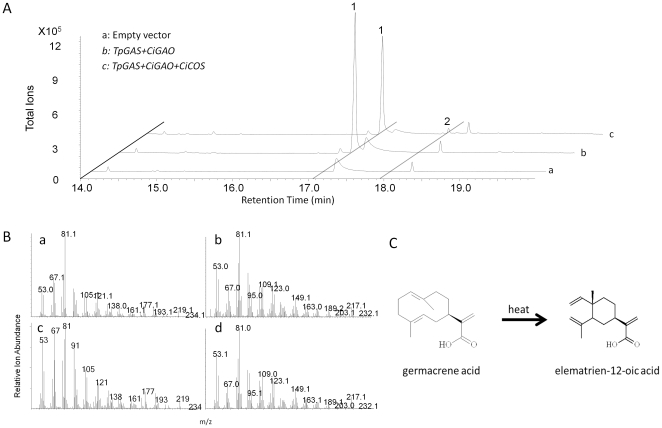
Costunolide production in yeast. A, GC-MS chromatograms are shown for the metabolites from yeast transformed with the indicated genes. Line a is a negative control, line b displays the metabolites in the yeast transformed with two genes (*TpGAS*, *CiGAO*), and line c displays the metabolites in the yeast transformed with three genes (*TpGAS*, *CiGAO*, and *CiCOS*). B, the mass spectra of compound 1 (a) and compound 2 (b) produced by yeast and elematrien-12-oic acid (c) and costunolide standards (d) are shown. C, cope rearrangement of germacrene acid to elematrien-12-oic acid by heat is shown. 1 = germacra-1(10),4,11(13)-trien-12-oic acid; 2 = costunolide.

Previously the chicory *germacrene A oxidase* (*CiGAO*, GenBank: GU256644) was characterized by expression in yeast [25]. We amplified the same gene (*CiGAO*) from a chicory cDNA library and the enzymatic activity was confirmed in our yeast system by co-expression of *TpGAS* and *CiGAO* (Figure 4A, line b). To test the activity of *CiGAO in planta*, an expression vector containing *CiGAO* was co-infiltrated with the *mTpGAS* expression vector into *N. benthamiana* leaves. In the headspace of *mTpGAS+CiGAO* agro-infiltrated *N. benthamiana* leaves, the germacrene A peak was no longer detected (compare Figure 3A line c with line d), suggesting that CiGAO can efficiently catalyse the conversion of the product of *mTpGAS*, germacrene A, into one or more other products. However, new peaks were visible neither in the headspace (Figure 3), nor in dichloromethane (DCM) extracts (data not shown) of *mTpGAS+CiGAO* agro-infiltrated *N. benthamiana* leaves compared with those of *mTpGAS*. Agro-infiltrated with *CiGAO* alone in *N. benthamiana* leaves, used as negative control, does not induce any volatile formation (compare Figure 3 line e and line d).

To investigate whether any non-volatile products were formed, the infiltrated leaves were extracted with aqueous methanol and the extracts analysed by accurate mass LC-QTOF-MS analysis. Metabolite profiles of the *pBIN* (empty vector), *mTpGAS*, and *mTpGAS+CiGAO* samples were recorded and the mass signals extracted in an untargeted manner using Metalign software (www.metAlign.nl), followed by clustering of extracted mass features into reconstructed metabolites [29]. The comparison of the *mTpGAS+CiGAO* co-infiltrated leaves and the *mTpGAS* infiltrated leaves revealed that, of the 2023 individual mass peaks with a signal-to-noise ratio higher than 3, none differed more than 2-fold (p<0.01, n = 3; student T-test) between the two sample groups. This result could be explained by conjugation of the expected products of GAO oxidation of germacrene A (germacra-1(10),4,11(13)-trien-12-ol, germacra-1(10),4,11(13)-trien-12-al and germacra-1(10),4,11(13)-trien-12-oic acid) to multiple compounds, resulting in a distribution of the product signal over multiple masses that apparently remain below the 2-fold threshold or the level of detection.

### Characterisation of a costunolide synthase gene from chicory

**Figure 5:**
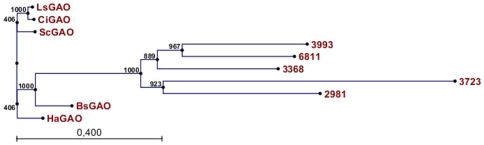
Phylogenetic analysis of Asteraceae GAO genes and five chicory CYP71 P450 ESTs. Chicory candidate 3368 was later identified as *Cichorium intybus costunolide synthase* (*CiCOS*). Amino acid seuqences of *GAOs* were obtained from cDNAs deposited at the NCBI. *LsGAO* germacrene A oxidase from *Lactuca sativa* (GU198171) or from *Cichorium intybus* (*Ci*; GU256644), *Helianthus annuus* (*Ha*; GU256646), *Saussurea costus* (*Sc*; GU256645) and *Barnadesia. spinosa* (*Bs*; GU256647). Bootstrap values are shown in frequency values from 1000 replicates.

Chicory (*Cichorium intybus* L.) accumulates costunolide in roots [8]. We assumed costunolide synthase to be a cytochrome P450 enzyme (as demonstrated by de Kraker *et al*, [6]) which evolved from the GAO gene and therefore should show close homology to the *CiGAO* amino acid sequences. A root-specific cDNA library from chicory was available [26], the sequences from the library were combined with chicory ESTs from GenBank (http://www.ncbi.nlm.nih.gov) and UC Davis database (http://compgenomics.ucdavis.edu/compositae_index.php), and these were searched for sequences with homology to the cytochrome P450 sequences of germacrene A oxidase from *Cichorium intybus* [*25]*, [26**] and *Lactuca sativa* [25]. Five P450 sequences were identified which clustered into class CYP71 and had high similarity to the *GAO* genes mentioned above. Figure 5 shows the phylogenetic relationship of the candidate chicory CYP71 P450 sequences and GAO genes from different plant species. Each of these candidate cDNA sequences was then cloned into a yeast expression vector and tested in the yeast expression system by co-transformation with *TpGAS* and *CiGAO*. One of the isolated cDNAs (3368) encodes an enzymatic activity which was able to produce costunolide (Figure 4) in the presence of *TpGAS* and *CiGAO*, and therefore was designated as *costunolide synthase* (*CiCOS*) (CYP71BL3).

As the conversion of the germacra-1(10),4,11(13)-trien-12-oic acid to costunolide was quite low in HEPES buffer (Figure 4), we tested the effect of another buffer, MOPS, on costunolide production in yeast, using UPLC-MRM MS to quantify costunolide production. For *TpGAS+CiGAO+CiCOS* transformed yeast cultured in HEPES buffer (pH 7.5) the costunolide production was 9 µg mL^−1^ culture, while in MOPS buffer (pH 7.5) production was about 3-fold higher (28 µg mL^−1^ culture).

To test the activity of the newly identified *CiCOS in planta*, the cDNA was cloned into a binary expression vector under control of the Rubisco promoter. *N. benthamiana* leaves were co-infiltrated with agrobacterium cultures with *RBC*::*mTpGAS*, *RBC::CiGAO* and *RBC::CiCOS* and after 3 days leaves were extracted with methanol and extracts were analysed by UPLC-MRM MS for quantification of free costunolide. Results show that average production of costunolide from eight infiltration experiments was 48.6±13.4 ng g^−1^ FW. No costunolide was detected in extracts from leaves infiltrated with either empty vector (pBIN), *RBC*::*mTpGAS*, *RBC*::*mTpGAS+RBC::CiGAO* or *RBC*::*mTpGAS+RBC::CiCOS* (data not shown), indicating that the production of costunolide by *CiCOS* in *N. benthamiana* leaves is dependent on the presence of both *TpGAS* and *CiGAO*.

**Figure 6:**
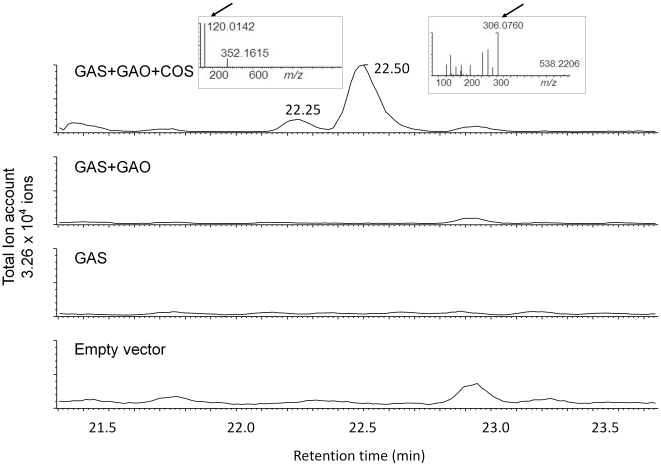
LC-MS/MS analysis of non-volatile metabolites in *N. benthamiana* leaves agro-infiltrated with empty vector, *TpGAS*, *TpGAS+CiGAO* and *TpGAS+CiGAO+CiCOS*. The two new peaks in *TpGAS+CiGAO+CiCOS a*gro-infiltrated leaves were further fragmented by MS/MS. The [*m/z*]^−^ for the parent ion of peak 22.30 is 352.1615 The [*m/z*]^−^ for the parent ion of peak 22.50 is 538.2206. Inserted figures show MS/MS spectrum of peak 22.30 and peak 22.50 at 25 eV. Arrows indicate characteristic Cys and GSH MS/MS fragments, respectively. The Y-axis scale is identical in all chromatograms.

To investigate whether there were any other metabolic changes caused by co-infiltration of *RBC*::*mTpGAS*, *RBC*::*CiGAO* and *RBC*::*CiCOS*, an untargeted LC-QTOF-MS analysis of aqueous methanol extracts from leaves was carried out. Comparison of the chromatograms of extracts from co-infiltrated leaves showed two new compounds, eluting at 22.30 and 22.48 min, in the leaves infiltrated with *mTpGAS+CiGAO+CiCOS* compared to leaves infiltrated with *mTpGAS+CiGAO* (Figure 6)

### Identification of costunolide conjugates

In order to identify the two new compounds in the leaves infiltrated with *mTpGAS+CiGAO+CiCOS*, the apparent parent masses of the peaks at 22.30 and 22.48 min were fragmented by LC-MS/MS in negative mode. Within the MS/MS fragments of 352.1615 (parent ion of peak at 22.30 min, a 9.2 ppm deviation from the elemental formula C_18_H_27_NO_4_S), we detected an ion with mass 120.0142, a 19 ppm deviation from the elemental formula of cysteine (C_3_H_7_NO_2_S). This MS/MS experiment therefore suggests that the peak at 22.30 min is a costunolide (C_15_H_20_O_2_)-cysteine (C_3_H_7_NO_2_S) conjugate. Within the MS/MS fragments of 538.2206 (parent ion of peak at 22.48 min, a −19.9 ppm deviation from the elemental formula C_25_H_37_N3O_8_S), we detected an ion with mass 306.0760, a −2.9 ppm deviation from the elemental formula of glutathione (C_10_H_17_N_3_O_6_S). This MS/MS experiment therefore suggests that the peak at 22.48 min is a costunolide (C_15_H_20_O_2_)-glutathione (C_10_H_17_N_3_O_6_S) conjugate (Figure 6).

**Figure 7:**
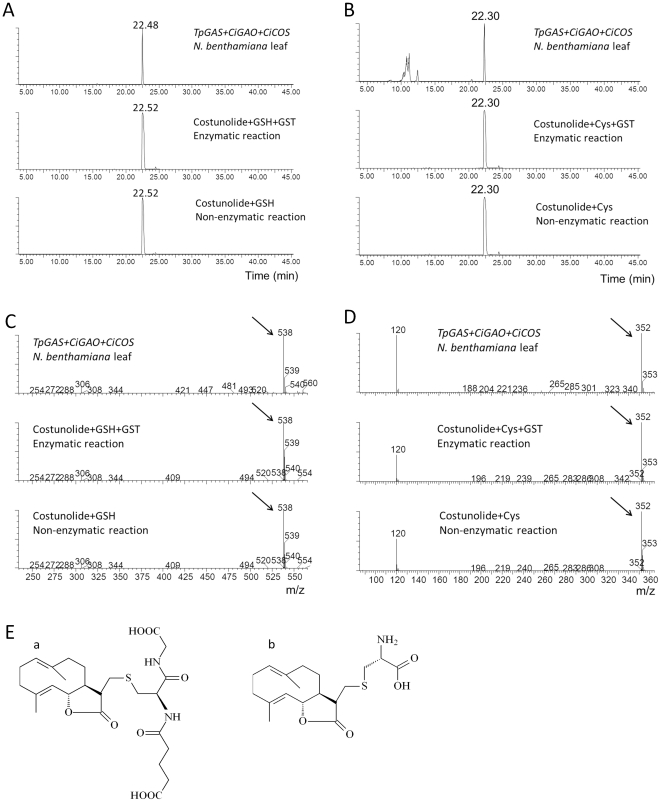
Costunolide-glutathione and costunolide-cysteine conjugate identification by GST enzyme assay and LC-MS analysis. A. LC-MS chromatograms of [*m/z*]^−^ = 538 of extracts of *N. benthamiana* leaves agro-infiltrated with *TpGAS+CiGAO+CiCOS*, costunolide-GSH conjugate formed in an enzyme assay of costunolide and GSH with GST, costunolide-GSH conjugate formed by non-enzymatic conjugation of costunolide and GSH. B. LC-MS chromatograms of [*m/z*]^−^ = 352 of extracts of *N. benthamiana* leaves agro-infiltrated with *TpGAS+CiGAO+CiCOS*, costunolide-cysteine (Cys) conjugate formed in an enzyme assay of costunolide and Cys with GST, costunolide-Cys conjugate formed non enzymatically from costunolide and Cys. C. [*m/z*]^−^ spectrum of peak 22.48 and costunolide-GSH conjugate (RT = 22.52). Arrows indicate parent ions of GSH-glutathione. D. [*m/z*]^−^ spectrum of peak 22.30 and costunolide-Cys conjugate (RT = 22.30). Arrows indicate parent ions of GSH-cysteine. E. Presumed molecular structure of costunolide-GSH (a) and costunolide-Cys (b) conjugates. GSH, glutathione; Cys, cysteine; GST, glutathione S-transferase. RT, retention time. Y-axis scale is identical in all chromatograms.

To further confirm the identity of these putative costunolide glutathione and cysteine conjugates, we tested the activity of a glutathione-S-transferase (GST) enzyme on costunolide in an *in vitro* enzyme assay. Analysis of the reaction mix of costunolide with glutathione and GST by LC-QTOF-MS showed that costunolide was efficiently converted into a costunolide-glutathione conjugate (Figure 7) that had the same retention time and exact mass and MS fragments as the postulated costunolide conjugate detected in the extract of the *mTpGAS+CiGAO+CiCOS* agro-infiltrated leaf sample (Figure 7A and 7C). When costunolide and glutathione were incubated without GST enzyme, the same costunolide-glutathione conjugate was formed indicating that the conjugation of costunolide and glutathione can occur spontaneously. Similarly, when costunolide was incubated with cysteine a costunolide-cysteine conjugate was spontaneously formed (Figure 7B). The cysteine conjugate that was spontaneously formed by the *in vitro* reaction had the same mass spectrum and retention time as the compound produced in *mTpGAS+CiGAO+CiCOS* agro-infiltrated *N. benthamiana* leaves (Figure 7B and 7D). Thus the two new peaks in *mTpGAS+CiGAO+CiCOS* agro-infiltrated *N. benthamiana* leave were confirmed to be a costunolide-glutathione and a costunolide-cysteine conjugate. The presumed structure of these two compounds is shown in Figure 7E.

## Discussion

### Reconstitution of the costunolide biosynthesis pathway in yeast

Costunolide is the precursor of many biologically active SLs, and reconstitution of its biosynthetic pathway in heterologous hosts could form an attractive option for commercial production of these compounds. Partial reconstruction of the sesquiterpene biosynthesis pathways in yeast has been demonstrated for the antimalarial drug artemisinin [30] and for germacra-1(10),4,11(13)-trien-12-oic acid [25], [26]. Here we reconstituted the biosynthetic pathway of costunolide in yeast and *in planta*. To achieve this we screened five candidate CYP71 P450 genes from chicory for costunolide synthase activity, which yielded one gene which had this activity, *CiCOS*. This novel gene was combined with a new *GAS* gene from feverfew (*TpGAS*) and the previously identified chicory *GAO* [25], [26]. Co-expression of *TpGAS* together with *CiGAO* and *CiCOS* in yeast yielded just low levels of costunolide, so it was of interest to see if production in yeast could be boosted. We showed that using GAS from different sources may have a strong effect on germacrene A production (Figure 2).

Also the culture buffer conditions strongly affect costunolide production. We showed that germacra-1(10),4,11(13)-trien-12-oic acid can be more efficiently converted by CiCOS into costunolide when the yeast is cultured in MOPS (pH 7.5) buffer instead of HEPES buffer (pH 7.5). The pH of the buffered yeast culture decreased from 7.5 to 6.8 for both MOPS and HEPES buffered culture after 48 hours of cultivation. Therefore, the difference in costunolide production was not due to the buffering capacity of the buffer. We presume that the increased costunolide production results from improved growth of yeast in the presence of MOPS, compared to the HEPES-buffered yeast culture.

### Reconstitution of the costunolide biosynthesis pathway in *N. benthamiana*

Reconstruction of pathways in the transient *Nicotiana* spp plant expression system was demonstrated for many medically relevant proteins [31] and has been shown to be a good model to study the production of sesquiterpenoid pharmaceutical compounds [27]. A marked peak of germacrene A was detected in the headspace of *mTpGAS* agro-infiltrated *N. benthamiana* leaves. The targeting of sesquiterpene synthases in metabolic engineering seems to have a great effect on its performance in plants. It has been shown in Arabidopsis that fusion of nerolidol synthase from strawberry to a mitochondrial targeting sequence leads to the biosynthesis of nerolidol, whereas this was not the case when using a cytosol targeting sequence [28]. This is in line with our observation that the germacrene A emitted from *N. benthamiana* leaves infiltrated with mitochondrial targeted germacrene A synthase is much higher than that from leaves infiltrated with the cytosolic germacrene A synthase (Figure 3).

The disappearance of germacrene A observed upon co-expression of mitochondrial *TpGAS* and *CiGAO* suggests that germacrene A is efficiently transferred from the mitochondrial compartment to the cytosol/ER, where it is presumably converted into germacrene A acid by CiGAO. The activity of mitochondrial *TpGAS* might produce a concentration gradient sufficient to drive diffusion of germacrene A into the cytosol. Or, as suggested by Turner and Croteau [32], some type of terpenoid carrier protein, mitochondrial membrane pump, or transient contacts between ER and mitochondrial membranes might facilitate germacrene A movement from mitochondria to cytosol/ER. Germacrene A could not be detected in the headspace or solvent extracts of *mTpGAS+CiGAO* agro-infiltrated *N. benthamiana* leaves. We suppose that this is due to an efficient conversion of germacrene A into germacra-1(10),4,11(13)-trien-12-oic acid by the expressed CiGAO enzyme. The fact that we were not able to detect germacra-1(10),4,11(13)-trien-12-oic acid may be explained by assuming that the compounds produced by CiGAO subsequently react with endogenous metabolites to form conjugates that were either not detected by our GC-MS and LC-QTOF MS (ESI negative mode) profiling approaches, or were converted and diluted out into many new metabolites of which the signals were below our detection levels. The disappearance of germacrene A after introduction of *CiGAO* suggests an efficient transfer of substrate between GAS and GAO. Similar results have been reported by van Herpen *et al.* [27]: co-infiltration of *N. benthamiana* with the cDNA encoding amorphadiene synthase plus *CYP71AV1*, also a sesquiterpene oxidizing P450, leads to an almost complete conversion of amorphadiene. Also in the latter paper, the product of this conversion, artemisinic acid was efficiently further converted to a diglucose conjugate [27].

Co-expression of *TpGAS* together with *CiGAO* and *CiCOS* in *N. benthamiana* leaves yielded up to 60 ng g^−1^ FW of costunolide. In addition, two novel costunolide conjugates were detected.

### Costunolide glutathione conjugates are present *in planta* but not in yeast

Most glutathione conjugations are catalysed by glutathione S-transferase (GST), which may be constitutively active or be induced upon oxidative stress or exogenous heterocyclic compounds, such as herbicides [33]. The expression of two glutathione S-transferases (*NbGSTU1* and *NbGSTU3*) in *N. benthamiana* was up-regulated progressively during infection by the fungus *Colletotrichum destructivum* [34]. Here we show that glutathione conjugates may be formed spontaneously from costunolide and GSH in an *in vitro* enzyme assay (Figure 7). S-glutathionylated metabolites are likely tagged for vacuolar import by ATP binding cassette (ABC) transporters, which selectively transport GSH conjugates, as has been shown for other glutathione S-conjugates [35]. Storage of target metabolites as glutathione S-conjugates may have the advantage that the storage capacity of the vacuole is used and that high concentrations can be reached without phytotoxic effects. Marrs *et al.* [36] reported that anthocyanin pigments require GSH conjugation for transport into the vacuole. If conjugation is inhibited, this leads to inappropriate cytoplasmic retention of the pigment which is toxic for the cells.

In addition to costunolide-GSH, costunolide-cysteine was also found to accumulate in agro-infiltrated *N. benthamiana* leaves. This cysteine conjugate may be a breakdown product of costunolide-glutathione [37]. However, we showed that the costunolide-cysteine conjugate may also be formed spontaneously from costunolide and cysteine (Figure 7).

Remarkably, no costunolide-glutathione or costunolide-cysteine conjugates were detected in medium of yeast transformed by *TpGAS+CiGAO+CiCOS* (data not shown), even though GSH is present in yeast [38] and some transporter genes, such as Bpt1p [39], have been reported to mediate vacuolar sequestration of glutathione conjugates in yeast. It could be that free costunolide is excreted out of the yeast cells and that any costunolide-glutathione conjugate formed inside the cell has a short half-life.

### Spontaneous conjugation to glutathione related to bioactivity of costunolide?

It has been shown that the effect of costunolide treatment of cancer cells is based on a rapid depletion of the intracellular reduced glutathione and protein thiols, which precedes apoptosis. Indeed, the effect of costunolide can be blocked by pretreatment with sulfhydryl compounds such as GSH, N-acetyl-L-cysteine, dithiothreitol and 2-mercaptoethanol [40]. The apoptosis-inducing activity of costunolide likely depends on the exomethylene moiety because derivatives in which this group was reduced, such as dihydrocostunolide and saussurea lactone, did not deplete the cellular thiols and showed no apoptotic activity [40], [41]. If the biological activity of costunolide depends on the ability to conjugate glutathione and thiols, then the costunolide-glutathione conjugate produced in *N. benthamiana* may not exhibit biological activity. On the other hand, the poor water-solubility of costunolide may also limit its potential as a promising clinical agent [42] and conjugation could improve this property. Regulation of conjugation in heterologous plant hosts and secretion into cell compartments that allow accumulation of free costunolide could therefore be an important target for further optimization of a costunolide production platform.

### In conclusion

We describe here the discovery of a new gene, *CiCOS*, and its functional characterization in yeast as well as *in planta*. *CiCOS* encodes the enzyme catalyzing the formation of a sesquiterpene lactone, costunolide, a promising anti-cancer medicine, and a crucial intermediate in the biosynthesis of many other sesquiterpene lactones with important biological activities, such as parthenolide. The cloning of this gene allows for the development of platforms – in microbial as well as in plant hosts – for the production of sesquiterpene lactones that can potentially be developed into new drugs. The conjugation of costunolide to glutathione and cysteine detected upon *in planta* expression has never been reported before in plants and could present new opportunities for high production because of better storability as well as for the development of drugs with better water solubility.

## Materials and Methods

### Isolation and cloning of costunolide synthase candidate gene from chicory

A previously reported chicory cDNA taproot library [26] was used for gene isolation. Five candidate cytochrome P450 contigs belonging to the CYP71 family were identified by sequence homology to known sesquiterpene monooxygenases. RACE PCR (Clontech) was used to obtain the sequence of the 5′- and 3′-region of the candidate contigs. Full length cDNAs of candidate genes were amplified from chicory cDNA with the addition of NotI/PacI restriction sites. They were subsequently cloned to the yeast expression vector pYEDP60 [43], which was modified to contain PacI/ NotI sites at the polylinker, and sequenced. The DNA sequence for the *chicory costunolide synthase* (*CiCOS*), has been deposited in GenBank under the accession number JF816041. The sequence was also submitted to David Nelson's cytochrome P450 homepage (http://drnelson.uthsc.edu/cytochromeP450.html) and was assigned the name CYP71BL3 [44].

### Plasmid construction for gene expression in yeast

For the production of germacra-1(10),4,11(13)-trien-12-oic acid in yeast, *CiGAO* and *TpGAS* genes were both cloned into the pESC-Trp yeast expression vector (Agilent technologies) with the *TRP1* auxotrophic selection marker. *CiGAO* [26] was subcloned from the yeast vector PYEDP60 [43] to the pESC-Trp vector using NotI/PacI restriction sites. The obtained construct was named *CiGAO* pESC-Trp. Subsequently, *TpGAS* was amplified from the pACYCDuet™-1 vector [23] using high fidelity Phusion polymerase (Finnzymes) with the addition of BamHI/KpnI restriction sites. The amplified product was digested by BamHI/KpnI and ligated into the *CiGAO* pESC-Trp plasmid, yielding the final plasmid *TpGAS*+*CiGAO* pESC-Trp. No terminal tags were added in these constructs. This plasmid was transformed into the WAT11 [45] yeast strain and the clones were selected on Synthetic Dextrose (SD) minimal medium (0.67% Difco yeast nitrogen base medium without amino acids, 2% D-glucose, 2% agar) supplemented with amino acids, but omitting L-tryptophane for auxotrophic selection of transformants. *TpGAS*+*CiGAO* pESC-Trp and pYEDP60 plasmids containing costunolide synthase candidates were co-transformed into the WAT11 yeast strain. After transformation yeast clones containing both plasmids were selected on SD minimal medium supplemented with amino acids, but omitting uracil, adenine sulphate and L-tryptophane for auxotrophic selection of transformants.

### Gene induction in yeast and metabolite extraction

For the induction of gene expression in yeast, the transformed WAT11 yeast strain with *TpGAS*+*CiGAO* pESC-Trp or co-transformed with *TpGAS*+*CiGAO* pESC-Trp and costunolide synthase candidate-PYED60-Ura-Ade were inoculated in 3 mL Synthetic Galactose (SG) minimal medium (0.67% Difco yeast nitrogen base medium without amino acids, 2% D-galactose, 2% agar) but omitting TRP or Trp-Ura-Ade amino acids, respectively. The yeast was cultured overnight at 30°C and 300 rpm. The start culture was diluted to OD 0.05 in SG minimal medium omitting Trp or Trp-Ura-Ade amino acids, respectively. All yeast induction experiments were performed in triplicates in 50 mL of culture volume. Cultures were buffered at pH 7.5 using 100 mM HEPES or 100 mM MOPS. After fermentation for 48 h at 30°C and 300 rpm, the medium was extracted with 20 mL ethyl acetate. From this, a 10 mL sample was taken and the ethyl acetate evaporated with a stream of N_2_ to a final volume of 1 mL which was analyzed by GC-MS. For UPLC- MRM-MS analysis the ethyl acetate in a 10 mL subsample was completely evaporated and the residue redissolved in 300 µl of 25% acetonitrile in water.

### Plasmid construction for expression in *Nicotiana benthamiana*

For expression in *N. benthamiana*, *TpGAS*, *CiGAO* and *CiCOS* were cloned into ImpactVector1.1 (http://www.impactvector.com/) to express them under the control of the Rubisco (RBC) promoter [46]. *TpGAS* was also cloned into ImpactVector1.5 to fuse it with the RBC promoter and the CoxIV mitochondrial targeting sequence. An LR reaction (Gateway-LR Clonase TM II) was carried out to clone each gene into pBinPlus binary [47] vector between the right and left borders of the T-DNA for plant transformation.

### Transient expression in *Nicotiana benthamiana*

*A. tumefaciens* infiltration (agro-infiltration) was performed according to the description of van Herpen *et al.* [*27*]. *A. tumefaciens* batches were grown at 28°C at 220 rpm for 24 hours in YEP media with kanamycin (50 mg L^−1^) and rifampicillin (34 mg L^−1^). Cells were harvested by centrifugation for 20 min at 4000×*g* and 20°C and then resuspended in 10 mM MES buffer containing 10 mM MgCl_2_ and 100 µM acetosyringone (4′-hydroxy-3′,5′-dimethoxyacetophenone, Sigma) to a final OD_600_ of ∼0.5, followed by incubation at room temperature under gentle shaking at 50 rpm for 150 min. For co-infiltration, equal volumes of the *Agrobacterium* batches were mixed. Batch mixtures were infiltrated into leaves of three-week-old *N. benthamiana* plants by pressing a 1 mL syringe without metal needle against the abaxial side of the leaf and slowly injecting the bacterium suspension into the leaf. *N. benthamiana* plants were grown from seeds on soil in the greenhouse with a minimum of 16 hour light. Day temperatures were approximately 28°C, night temperatures 25°C. After agro-infiltration the plants were grown under greenhouse conditions for another 3 days and then harvested for analysis.

### Headspace analysis and GC-MS thermodesorption

Volatile collection from agro-infiltrated *N. benthamiana* leaves and GC-MS analysis were performed according to van Herpen *et al.* [27]. Steel sorbent cartridges (89 mm×6.4 mm O.D.; Markes) containing Tenax were used for volatile collection. Cartridges were conditioned at 280°C for 40 min under a nitrogen flow of 20 psi in a TC-20 multi-tube conditioner and were capped airtight until use. *N. benthamiana* leaves were sampled and placed on water in a small vial and were enclosed in a glass container. To trap the leaf-produced volatiles, air was sucked through the containers with a flow rate of 90 mL min^−1^ for 24 hours and released through one cartridge. A second cartridge was used to purify the incoming air. Sample cartridges were dried for 15 min at room temperature with a nitrogen flow of 20 psi before GC-MS analysis on a Thermo Trace GC Ultra connected to a Thermo Trace DSQ quadruple mass spectrometer (Thermo Fisher Scientific, USA).

Cartridges were placed in an automated thermodesorption unit (Ultra; Markes, Llantrisant) in which they were flushed with helium at 50 mL min−1 for 2 min to remove moisture and oxygen just before thermodesorption. The volatiles were desorbed by heating of the cartridges at 220°C for 5 min with a helium flow of 50 mL min^−1^. The compounds released were trapped on an electrically cooled sorbent trap (Unity; Markes, Llantrisant) at a temperature of 5°C. Subsequently, the trapped volatiles were injected on the analytical column (ZB-5MSI, 30 m×0.25 mm ID, 1.0 µm – film thickness, Zebron, Phenomenex) in splitless mode by ballistic heating of the cold trap to 250°C for 3 min. The temperature program of the GC started at 40°C (3 min hold) and rose 10°C min^−1^ to 280°C (2 min hold). The column effluent was ionised by electron impact (EI) ionisation at 70 eV. Mass scanning was done from 33 to 280 *m/z* with a scan time of 4.2 scans s^−1^. Xcalibur software (Thermo, USA) was used to identify the eluted compounds by comparing the mass spectra with those of authentic reference standards.

### GC-MS analysis of solvent extracts

Seven mL yeast culture was extracted three times with 2 mL ethyl acetate, which was concentrated, dried using anhydrous Na_2_SO_4_ and used for GC-MS analysis. Agro-infiltrated leaves (100 mg) were ground in liquid nitrogen and extracted with 800 µl dichloromethane. The extracts were prepared by brief vortexing and sonication for 10 min. Then the extracts were centrifuged for 15 min at 3000 rmp, dehydrated using Na_2_SO_4_, and then used for GC-MS analysis. A gas chromatograph (7809A, Agilent, USA) equipped with a 30 m×0.25 mm, 0.25 mm film thickness column (ZB-5, Phenomenex) using helium as carrier gas at flow rate of 1 mL min^−1^ was used for GC-MS analysis. Splitless mode was used for the injector with the inlet temperature set to 250°C. The initial oven temperature was 45°C for 1 min, and was increased to 300°C after 1 min at a rate of 10°C min^−1^ and held for 5 min at 300°C. The GC was coupled to a Triple-Axis detector (5975C, Agilent). Compounds were identified by comparison of mass spectra and retention times (RT) with those of the following authentic standards: germacrene A, germacra-1(10),4,11(13)-trien-12-ol, germacra-1(10),4,11 (13)-trien-12-al [26] and costunolide (TOCRIS bioscience). Quantification of sesquiterpenoids was conducted by determination of total ion count (TIC) peak area of the sesquiterpenoid peaks from three independent fermentation experiments. An absolute concentration of sesquiterpenoids was calculated from the peak area by comparison with calibration curves of the authentic standards. At the routine injection port temperature of 250°C germacrene A, germacra-1(10),4,11(13)-trien-12-oic acid and costunolide are thermally converted into β-elemene, elematrien-12-oic acid, and saussurea lactone, respectively as discussed by de Kraker et al (de Kraker et al., 2003; de Kraker et al., 1998; de Kraker et al., 2002). We also regularly injected samples with an injection port temperature of 150°C to confirm the presence of non-rearranged germacrene A, germacra-1(10),4,11(13)-trien-12-oic acid and costunolide.

### LC-QTOF MS and MS/MS analysis

Non-volatile metabolites were analysed by LC–QTOF-MS (liquid chromatography, coupled to quadrupole time-of-flight mass spectrometry) according to a protocol for untargeted metabolomics of plant tissues [48]. A Waters Alliance 2795 HPLC connected to a Waters 2996 PDA detector and subsequently a QTOF Ultima V4.00.00 mass spectrometer (Waters, MS technologies, UK) operating in negative ionization mode was used. An analytical column (Luna 3 µ C18/2 100A; 2.0×150 mm; Phenomenex, USA) attached to a C18 pre-column (2.0×4 mm; Phenomenex, USA) was used. Degassed eluent A [ultra-pure water: formic acid (1000∶1, v/v)] and eluent B [acetonitril∶formic acid (1000∶1, v/v)] were used at a flow rate of 0.19 mL min^−1^. Masses were recorded between m/z X and m/z Y; leucine enkaphalin ([M-H]^−^ = 554.2620) was used as a lock mass for on-line accurate mass correction.

For agro-infiltrated *N. benthamiana*, 100 mg infiltrated leaf from each treatment was ground in liquid nitrogen and extracted with 300 µl methanol∶formic acid (1000∶1, v/v). After brief vortexing and sonication for 15 min, the extracts were centrifuged for 5 min at 13,000 rpm and filtered through a 0.2 µm inorganic membrane filter (RC4, Sartorius, Germany). The gradient of the HPLC started at 5% eluent B and increased linearly to 75% eluent B in 45 min, after which the column was washed and equilibrated for 15 min before the next injection. The injection volume was 5 µl. Data-directed MS-MS measurements were done at collision energies of 10, 15, 25, 35 and 50 eV.

### Costunolide detection and quantification by UPLC- MRM- MS

Targeted analysis of costunolide in yeast extract and agro-infiltrated *N. benthamiana* leaves was performed by comparing retention times and mass transitions with that of a costunolide standard (TOCRIS bioscience) using ultraperformance liquid chromatography (UPLC) coupled to MS/MS essentially as described by Kohlen *et al.* [49] with some modifications. A Waters Xevo tandem quadrupole mass spectrometer equipped with an electrospray ionization source and coupled to an Acquity UPLC system (Waters) was used for analysis. Chromatographic separation was obtained on an Acquity UPLC BEH C18 column (150×2.1 mm, 1.7 µm; Waters) by applying a water/acetonitrile gradient to the column, starting from 5% (v/v) acetonitrile in water for 2.0 min and rising to 75% (v/v) acetonitrile in water in 45.0 min, followed by an increase to 90% (v/v) acetonitrile in water, which was maintained for 5.0 min before returning to 5% acetonitrile in water using a 0.2 min gradient, prior to the next run. Finally, the column was equilibrated for 2.8 min using this solvent composition. Operation temperature and flow rate of the column were 50°C and 0.4 mL min^−1^, respectively. Injection volume was 15 µL. The mass spectrometer was operated in positive electrospray ionization mode. Cone and desolvation gas flows were set to 50 and 1,000 L h^−1^, respectively. The capillary voltage was set at 3.0 kV, the source temperature at 150°C, and the desolvation temperature at 650°C. The cone voltage was optimized for costunolide using the Waters IntelliStart MS Console. Argon was used for fragmentation by collision-induced dissociation in the ScanWave collision cell. MRM was used for identification of costunolide in yeast extract and agro-infiltrated *N. benthamiana* leaves by comparing retention times and MRM mass transitions with that of a costunolide standard. MRM transitions were optimized for costunolide using the Waters IntelliStart MS Console.

### GC–MS and LC–MS data processing

GC-MS and LC-MS data analysis was done according to the description by Yang *et al.* (2011) [50] [49] [49] [49] [49] [50] [50] [50] with minor modifications. GC–MS data were acquired using Xcalibur 1.4 (Thermo Electron Corporation) and LC–MS data using MassLynx 4.0 (Waters). The data were processed using MetAlign version 1.0 (www.metAlign.nl) for baseline correction, noise elimination and subsequent spectral data alignment [48]. The processing parameters of MetAlign for GC–MS data were set to analyse scan numbers 1,340–16,000 (corresponding to retention times 2.32 to 28.05 min) with maximum amplitude of 1.4×10^8^. The processing parameters for LC–MS data were set to analyse scan numbers 60–2300 (corresponding to retention time 1.4 to 49.73 min) with a signal-to-noise ratio higher than 3.

To combine mass signals belonging to the same metabolite, all the detected masses were clustered by an in-house developed script called Multivariate Mass Spectra Reconstruction (MMSR) (Tikunov et al., 2005). The mass signal intensities (expressed as peak height using MetAlign) obtained from agro-infiltrated plants and empty vector control plants were compared using the Student's t-test. Masses with a significant (*p*<0.05) intensity change of at least 2-fold were verified manually in the original chromatograms.

To annotate significantly different compounds in LC-QTOF-MS, accurate masses were manually calculated, taking into account only those scans with the proper intensity ratios of analyte and lock mass [between 0.25- and 2 [51]] and elemental formulae generated within 5 ppm deviation from the observed mass. In addition, data-directed LC–MS/MS experiments were performed on differential compounds. To obtain proper MS/MS spectra only molecular ions with signal intensities higher than 500 ion counts per scan were selected.

### Costunolide conjugation enzyme assay

This enzyme assay was performed according to the method of Habig *et al.* [52] with modifications. In brief, glutathione (150 mM) or cysteine (150 mM) in 7 µl potassium buffer (100 mM; pH 6.5), and costunolide (30 mM) in 7 µl ethanol were added to 200 µl potassium buffer (100 mM; pH 6.5). The reaction was initiated by adding 7 µl of GST (1 g L^−1^, in 100 mM potassium buffer; pH 6.5) into the mixture. Complete assay mixtures without GST enzyme or either of the substrates were used as controls. After incubation for 15 min at room temperature, samples were kept at −20°C until analysis by LC-QTOF-MS.
